# Creating a more robust 5-hydroxymethylfurfural oxidase by combining computational predictions with a novel effective library design

**DOI:** 10.1186/s13068-018-1051-x

**Published:** 2018-03-01

**Authors:** Caterina Martin, Amaury Ovalle Maqueo, Hein J. Wijma, Marco W. Fraaije

**Affiliations:** Molecular Enzymology Group, Groningen Biomolecular Sciences and Biotechnology Institute, University of Groningen, Nijenborgh 4, 9747 AG Groningen, The Netherlands

**Keywords:** Enzyme engineering, Carbohydrate, Poly(ethylene-furandicarboxylate) PEF, Computational library, Golden Gate gene shuffling

## Abstract

**Background:**

HMF oxidase (HMFO) from *Methylovorus* sp. is a recently characterized flavoprotein oxidase. HMFO is a remarkable enzyme as it is able to oxidize 5-hydroxymethylfurfural (HMF) into 2,5-furandicarboxylic acid (FDCA): a catalytic cascade of three oxidation steps. Because HMF can be formed from fructose or other sugars and FDCA is a polymer building block, this enzyme has gained interest as an industrially relevant biocatalyst.

**Results:**

To increase the robustness of HMFO, a requirement for biotechnological applications, we decided to enhance its thermostability using the recently developed FRESCO method: a computational approach to identify thermostabilizing mutations in a protein structure. To make this approach even more effective, we now developed a new and facile gene shuffling approach to rapidly combine stabilizing mutations in a one-pot reaction. This allowed the identification of the optimal combination of seven beneficial mutations. The created thermostable HMFO mutant was further studied as a biocatalyst for the production of FDCA from HMF and was shown to perform significantly better than the original HMFO.

**Conclusions:**

The described new gene shuffling approach quickly discriminates stable and active multi-site variants. This makes it a very useful addition to FRESCO. The resulting thermostable HMFO variant tolerates the presence of cosolvents and also remained thermotolerant after introduction of additional mutations aimed at improving the catalytic activity. Due to its stability and catalytic efficiency, the final HMFO variant appears to be a promising candidate for industrial scale production of FDCA from HMF.

**Electronic supplementary material:**

The online version of this article (10.1186/s13068-018-1051-x) contains supplementary material, which is available to authorized users.

## Background

**Scheme 1:**
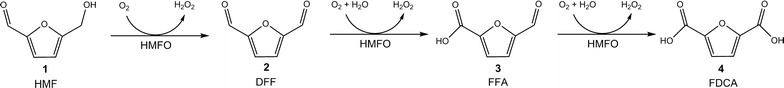
Oxidation reaction of 5-hydroxymethylfurfural (HMF) to 2,5-furandicarboxylic acid (FDCA) by HMFO

The solution to overcome the increasing demand for polymers, while fossil oil resources are rapidly depleting, could be the use of biomass as a renewable starting material [1]. A promising alternative to the petroleum-based poly(ethylene-terephthalate) (PET) polymer is poly(ethylene-furandicarboxylate) (PEF). Thanks to its similar characteristics, PEF has attracted attention, because it is a bio-based polymer obtained from furan-2,5-dicarboxylic acid (FDCA) and this monomer can be synthesized from the most abundant renewable material: carbohydrates [2, 3]. For this reason, FDCA production has gained a lot of interest and different methods have been developed to convert carbohydrates or carbohydrate derived products (such as HMF) into FDCA [4]. Chemical methods to obtain FDCA from HMF by homogeneous or supported metal catalysts typically present several disadvantages: high costs, low yields, byproducts, and the requirement of organic solvents which are not environmentally sustainable [5, 6]. Several biocatalytic routes from HMF to FDCA have recently been described [7–9]. Among these approaches, only one was shown to be dependent on merely one biocatalyst, HMF oxidase (HMFO), a highly attractive feature. We identified HMFO in the predicted proteome of *Methylovorus* sp. strain MP688 [8]. The oxidase contains a flavin adenine dinucleotide (FAD) cofactor as prosthetic group and only requires molecular oxygen as electron acceptor to catalyze various oxidation reactions which include alcohol and thiol oxidations [8, 10]. HMFO was shown to be capable to convert HMF into FDCA in a three-step reaction (Scheme 1) [8, 10]. To develop HMFO into an industrially applicable biocatalyst, its catalytic and stability properties have to be improved. While the enzyme is efficient in catalyzing the first step in the catalytic cascade, it is rather inefficient in the last step. Furthermore, the enzyme displays only a modest stability. Upon elucidating the crystal structure of HMFO, we successfully identified mutations that improve its performance in oxidation of the hydrated form of 5-formyl-2-furancarboxylic acid (FFA) to FDCA, the last step in the catalytic cascade [11]. The mutant V367R-W466F HMFO was found to be the best variant for FDCA production with a *k*_cat_/*K*_m_ value for FFA conversion > 1000-fold higher than for the wild-type enzyme [11]. Yet, as often observed when engineering enzyme activity, the higher catalytic performance was at the expense of enzyme thermostability.

Since HMFO has the potential to become an important biocatalytic tool, development of a robust variant is essential. An enzyme with a higher thermostability will be more suitable for use under harsh reaction conditions such as elevated temperatures and the presence of organic cosolvents [12]. Moreover, a stable enzyme is the ideal template for further enzyme engineering efforts aimed to improve or change its catalytic properties [13]. Several methods have been designed to enhance the (thermo)stability of enzymes [14]. Completely random approaches like directed evolution can be effective but are time-consuming and require high-throughput screening methods [15].

Rational design of stabilizing mutations is still challenging due to the fact that it is too complicated to accurately explain the effects of a mutation in terms of ΔΔH and ΔΔS [16]. Yet, state-of-the-art computational methods have evolved to such a level that they have predictive value in selecting putative thermostabilizing mutations. This can be used as input for the design of relatively small mutant libraries for screening in vitro [17]. We have recently developed a framework for rapid enzyme stabilization by computational libraries (FRESCO) which is a computationally assisted method that includes predicting a large number of independent stabilizing mutations that are in silico screened to define a relatively small set of mutations that need to be tested experimentally [18, 19]. This approach has demonstrated its validity with different enzymes, leading to high *T*_m_^app^ improvements of up to 35 °C [18, 20–22]. The initial steps of the FRESCO strategy consist of computational and visual selections based on free energy predictions and molecular dynamic (MD) simulations of single point mutations. This is followed by an in vitro phase to identify variants with significantly improved stability, and finally, the combination of the selected mutations should result in a highly stable enzyme variant. FRESCO is an attractive approach when dealing with a protein for which the crystal structure has been determined and, therefore, HMFO is a suitable candidate. The aim of this work was to obtain a stable HMFO variant which performs well in the conversion of HMF into FDCA.

## Results and discussion

### Computational screening

The in silico phase of the FRESCO strategy was necessary to create an enriched library of single point mutants of HMFO. The crystal structure of the reduced form of the enzyme (PDB:4UDQ) was selected as model as it was solved at a better resolution (1.6 Å) than the oxidized enzyme [11]. Using this structure, all possible point mutations were modeled (excluding residues that are within 5 Å from the FAD cofactor) and their respective values in free energy of folding were compared with that of the wild-type enzyme (∆∆G^Fold^). By omitting the residues that are close to the active site, the risk of creating a mutant with lower or no activity is limited. The first in silico step consisted of a selection based on free energy prediction: single mutants with a predicted ∆∆G^Fold^ higher than − 5 kJ mol^−1^ were discarded. This decreased the number of variants to screen from 9044 to 744. The dynamic disulfide discovery (DDD) algorithm was not included in this FRESCO approach, because disulfide bonds may complicate protein expression [22]. The MD simulations of the 744 variants were visually inspected comparing the modeled structure of the wild-type with the mutant ones. The goal was to select variants with putative improvements in their thermostability profile. The screening was based on avoiding features that are normally found to cause a decrease in stability such as an increased flexibility (backbone and sidechain), an increase in hydrophobic surface exposure and diminished hydrogen-bonding interactions [23]. Those 140 mutants that scored well in the free energy of folding and did not show such aberrant structural features upon MD simulation and visual inspection were selected to be experimentally tested for thermostability.

### Screening of single mutants

**Fig. 1:**
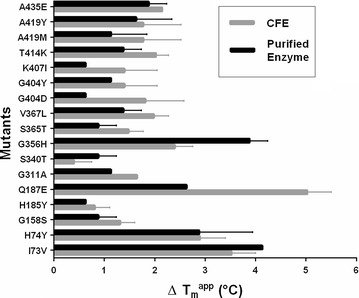
Δ*T*_m_^app^ values of 17 selected mutants. The values correspond to an increase in *T*_m_^app^ when compared with WT HMFO (*T*_m_^app^ = 48.5 °C)

Using PCR, all 140 mutations were introduced in the HMFO expression plasmid. The 140 mutant proteins and wild-type HMFO were produced in 96-well plates, which allowed efficient testing of all variants. To establish the effect of each mutation on stability, the apparent melting temperature (*T*_m_^app^) was determined for each variant using the ThermoFAD assay [24]. The latter method reports on the temperature at which the flavin cofactor is released from the protein, thereby becoming more fluorescent, because flavin fluorescence is typically quenched when it is bound in a protein. This provides a good estimate of the thermostability of the studied flavoprotein as cofactor release is the result of protein unfolding. Because HMFO is well expressed, we did not only determine the *T*_m_^app^ value for each variant after enzyme purification, but the *T*_m_^app^ was also measured using cell extracts. This revealed that the stability of the variants could also be measured in cell extracts as the *T*_m_^app^ values obtained with cell-free extracts were quite consistent compared with the purified enzymes. It demonstrates that the ThermoFAD method can be a very powerful approach to efficiently screen a large set of flavoprotein variants without the need for enzyme purification. Importantly, the ThermoFAD measurements revealed 17 mutations that led to a significant positive Δ*T*_m_^app^ in comparison with the wild-type enzyme (Fig. 1) (Additional file 1: Table S1).

### Golden Gate gene shuffling

**Fig. 2:**
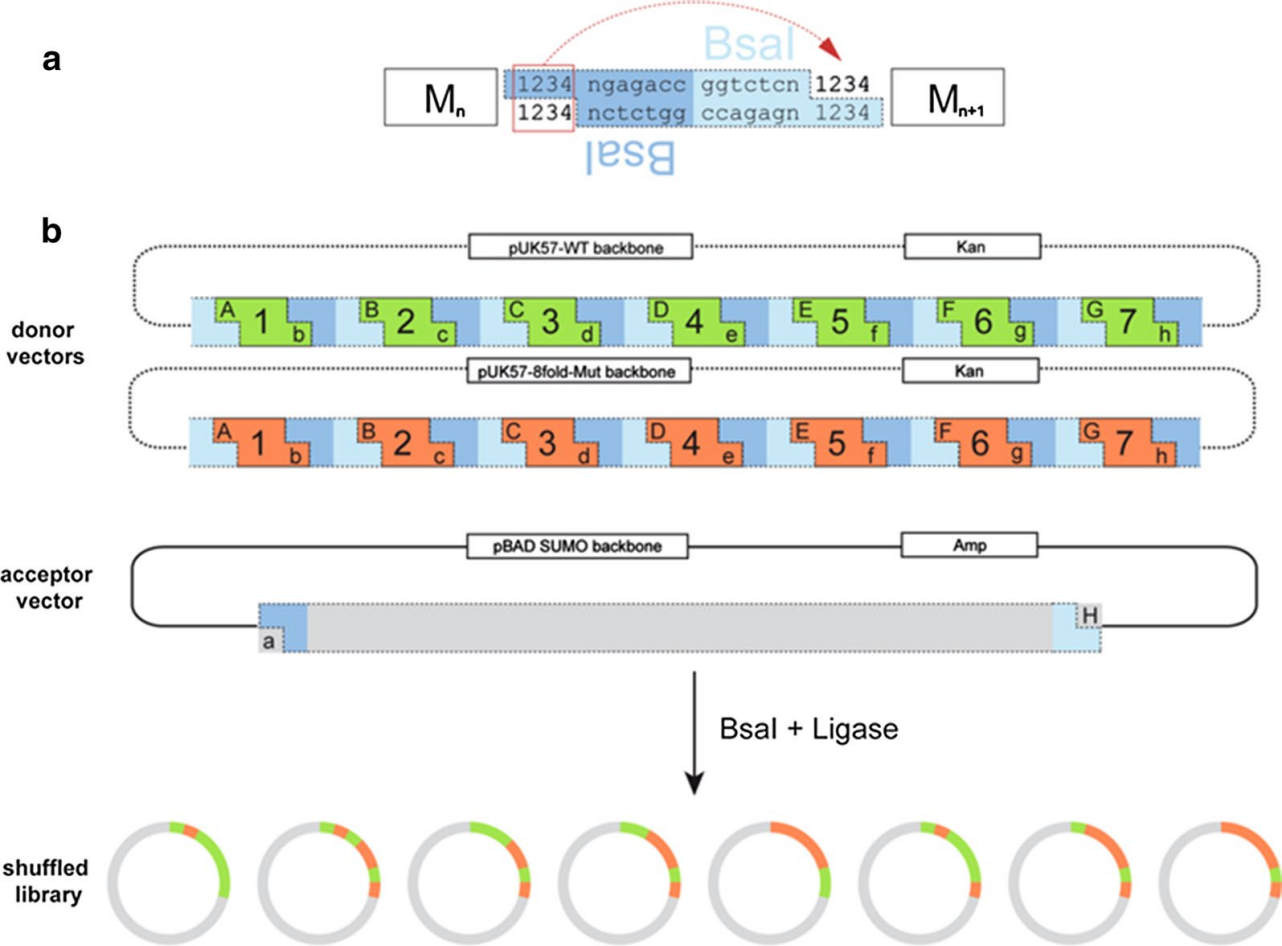
Design of the synthetic genes for Golden Gate gene shuffling. **a** BsaI recognition sites between two modules are flanked and mirrored. The four nucleotides at the end of one fragment are replicated at the beginning of the next one to avoid nucleotide loss after the ligation. **b** Donor vectors have the same fragment arrangement. The wild-type fragments are represented in green, while the fragments in orange have one mutation site each. The dotted sections (pUK57 backbone and BsaI sites) are lost after the ligation. The shuffled library consists of pBAD SUMO-HMFO vectors with a random combination and correct order of the 7 gene modules (with mutations)

As next step in the FRESCO method, the best combination of beneficial mutations needs to be identified. The addition of several single mutations does not necessarily lead to a more thermostable variant, because beneficial single mutations may not be compatible. So far, the strategy to combine the mutations was based on a good analysis of the Δ*T*_m_^app^ together with the mutation position and its predicted effect. Yet, such an approach is time-consuming, since the mutations need to be combined in a step-wise fashion, one by one or in groups and tested at each step [18, 20–22]. We set out to develop a more effective method to quickly identify the best combination of mutations that results in a stable and active variant. For this, we aimed at randomly combining mutations through a gene shuffling approach. Various gene shuffling methods have been developed in the field of protein engineering, often with the focus to merge certain wanted properties of two or more distinct proteins into one [25, 26]. We developed a controlled approach that can be applied in cases when it is desired to test all different combinations of several specific gene fragments (with predefined mutations). In our method, we take advantage of the fact that gene (fragment) synthesis has become relatively cheap [27]. For the gene shuffling library, we chose the eight mutations which resulted in the highest *T*_m_^app^ values: I73V, H74Y, Q187E, G356H, V367L, T414K, A419Y, and A435E. To assess if the catalytic properties of the corresponding mutant enzymes were un-affected, we measured HMFO activity towards vanillyl alcohol [8]. The benefit of using this substrate is that it allows direct detection of product formation as the formed vanillin displays light absorbance at 340 nm. Gratifyingly, all mutants showed similar activity when compared with the wild-type enzyme (Additional file 2: Table S2). Before performing gene shuffling, the mutations I73V and H74Y, being neighboring residues, were combined to verify whether they display an additive effect. This was found to be the case as the double mutant presents a *T*_m_^app^ that is 6 °C higher when compared with the wild-type enzyme (the single mutations displayed 3–4 °C higher *T*_m_^app^ values, Fig. 1). This double mutation was included in the gene shuffling approach instead of the two individual mutations, reducing the number of mutation sites to 7. The aim of the gene shuffling approach was to create a library of all the possible combinations (128) of the selected 7 sites and subsequently test them for thermostability and activity towards HMF. The gene shuffling protocol described in this work is based on the Golden Gate cloning system [28]. Previously, other approaches used type IIs restriction enzymes to combine gene fragments or plasmid modules; these methods involve cloning of each module flanked by BsaI sites in individual vectors, or add type IIs restriction enzyme sites by separate PCRs for each module [29, 30]. The Golden Gate gene shuffling which we developed for the FRESCO strategy is a one-pot reaction that involves only three designed vectors and a single restriction–ligation reaction (Fig. 2).

The first step was the design of two synthetic genes, one wild-type version and one with the selected 7 mutations; each containing 8 BsaI restriction sites that flank the 7 gene modules that contain the target mutation sites. The positions of the modules were defined identically for the two versions and were designed, such that all the desired mutation sites could be introduced in separate modules and that the four-nucleotide overhangs for the ligation were unique and not palindromic. The innovation of this method is in the design of the BsaI restriction sites. Each module is flanked by two BsaI sites: between two modules there are two mirrored BsaI sites and the four overhang nucleotides at the end of each module are replicated at the beginning of the following module to allow a scarless ligation. By performing the one-pot Golden Gate cloning reaction on the mixture of the two donor vectors, containing the two synthetic genes, and the acceptor pBAD vector, all possible combinations of the 7 gene fragments are created in the pBAD-based expression vector. The Golden Gate gene shuffling library was analyzed by sequencing to confirm the heterogeneity and accuracy of the shuffling of gene fragments. The results showed that 97% of the colonies contained the correct restriction pattern and the shuffling efficiency was 65%. This indicates that per 100 clones, 65 contain non-redundant randomly shuffled sequences.

### Shuffled library screening

The gene shuffling library was tested to establish *T*_m_^app^ of the variants and also their activity towards HMF. To be sure of screening all the possible combinations, the tested library size was 3.5-fold the number of possible combinations. The results of the ThermoFAD analysis showed that the 8xHMFO (I73V, H74Y, Q187E, G356H, V367L, T414K, A419Y, and A435E) was the most thermostable variant with an improvement of 13 °C compared with the WT (*T*_m_^app^ = 48.5 °C). Yet, an alignment of the best 9 multi-site mutants that presented an higher *T*_m_^app^ together with a preserved ability to oxidize HMF demonstrated that the mutation Q187E had a negative effect on the activity (Additional file 3: Table S3). This shows the advantage of using a gene shuffling approach to evaluate the best thermostable and active mutant. Therefore, the best multiple mutant was considered to be the 7xHMFO mutant (I73V, H74Y, G356H, V367L, T414K, A419Y, and A435E) with a *T*_m_^app^ of 60.5 °C and *k*_cat_ and *K*_m_ values comparable with the wild-type enzyme (Table 1) [8].

**Table 1:** Steady-state parameters of the different HMFO variants on HMF

HMFO	*k*_cat_ (s^−1^)	*K*_m_ (mM)	*k*_cat_/*K*_m_ (s^−1^ mM^−1^)
WT	13.7	1.52	9.03
7xHMFO	11.8	0.63	18.7

Kinetic parameters were determined by measuring H_2_O_2_ formation in a coupled assay using HMF as substrate (Additional file 4: Figure S1)

**Fig. 3:**
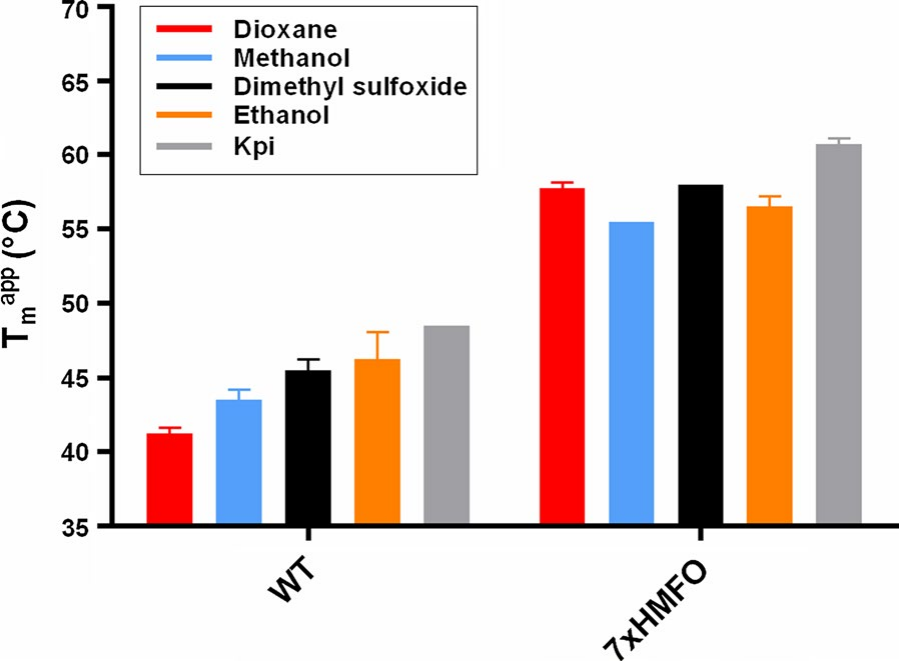
*T*_m_^app^ in the absence or presence of 15% 1,4-dioxane, methanol, dimethyl sulfoxide, or ethanol. Kpi is the control in 50 mM phosphate buffer, pH 8.0

To further investigate the thermostable properties of the 7xHMFO mutant, its stability over time at 40 °C was investigated. The 7xHMFO variant presented a remarkable stability compared to the wild-type enzyme. While wild-type HMFO completely lost its activity after 2 days, the thermostable variant retained 50% of its activity even after 10 days of incubation at 40 °C (Additional file 5: Figure S2). Since HMFO is able to oxidize many different substrates, including compounds that are poorly soluble in water, we investigated the stability in the presence of different cosolvents. The 7xHMFO showed a much higher tolerance towards four commonly used cosolvents when compared with the wild-type enzyme (Fig. 3). The 7xHMFO mutant with its higher thermostability, solvent-tolerance, and preserved catalytic activity represents an excellent template for further engineering.

### Engineering of thermostable HMFO variant for FDCA production

To further tune the 7xHMFO variant towards conversion of HMF into FDCA, we introduced two mutations (V367R and W466F) in the active site that have been shown to be beneficial for FDCA production from HMF [11]. Since V367 had already been mutated in the 7xHMFO variant (V367L), we included in the analysis also another 8xHMFO variant with only the W466F mutation. The variants were tested, and as expected, the additional mutation(s) introduced into the 7xHMFO decreased the *T*_m_^app^. Yet, the stability remained significantly higher than the wild-type enzyme (Table 2).

**Table 2:** *T*_m_^app^ values of HMFO variants

HMFO	*T*_m_^app^ (°C)
WT	48.5
V367R W466F	39.5
7xHMFO^a^	60.5
8AxHMFO^b^	55.5
8BxHMFO^c^	51.5

*T*_m_^app^ values of HMFO variants (30 μM) obtained by ThermoFAD in phosphate buffer 50 mM pH 8.0

^a^The 7xHMFO variant includes the mutations: I73V, H74Y, G356H, V367L, T414K, A419Y, and A435E

^b^The 8AxHMFO variant includes the mutations: I73V, H74Y, G356H, V367L, T414K, A419Y, A435E, and W466F

^c^The 8BxHMFO variant includes the mutations: I73V, H74Y, G356H, V367R, T414K, A419Y, A435E, and W466F

**Fig. 4:**
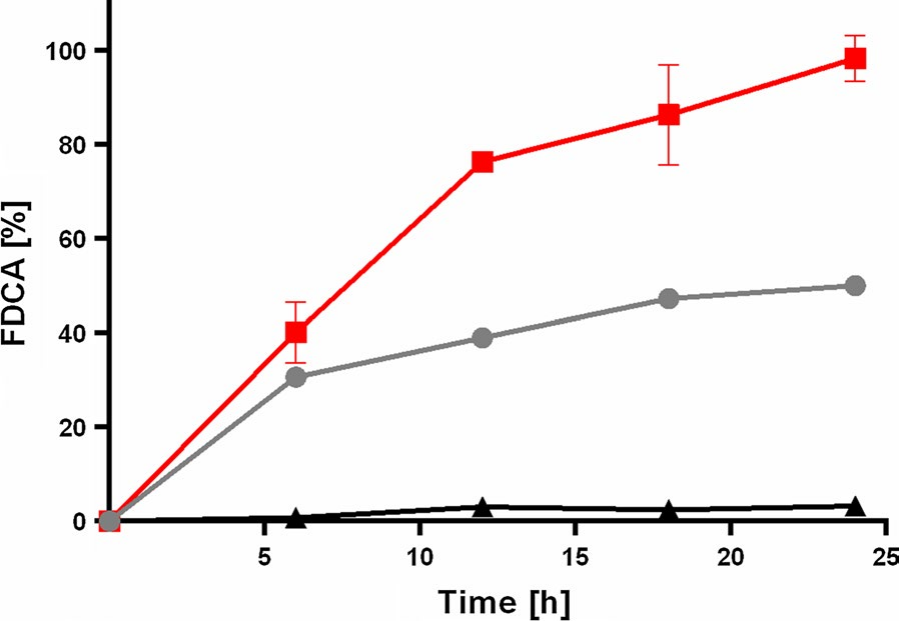
Production of FDCA from HMF at 25 °C by HMFO variants. WT (filled triangle), V367R W466F HMFO (filled circle), 8BxHMFO (I73V-H74Y, G356H, V367R, T414K, A419Y, A435E, and W466F) (filled square). Conversions were performed in duplicates in phosphate buffer 50 mM pH 8.0, HMFO 2.0 μM, HMF 5.0 mM, 25 °C

To determine whether the thermostable variants are more potent biocatalysts than the previously described wild-type and/or V367R W466F HMFO mutant, we tested the conversion rates using HMF as a substrate. The best thermostable variant for FDCA production turned out to be the newly engineered 8BxHMFO. The data revealed that this HMFO variant performs significantly better than the wild-type and the double mutant (Fig. 4) (Additional file 6: Table S4). At 25 °C, an almost full conversion in 24 h could be achieved, while the conversion with the double mutant and the wild-type enzyme remained below 50 and 5%, respectively. Since all the generated thermostable HMFO variants exhibit *T*_m_^app^ values higher than 50 °C, we decided to perform conversions also at 40 °C. At this temperature, only the 8BxHMFO mutant performed well (> 95% conversion in only 9 h) (Additional file 7: Table S5). The *k*_cat_/*K*_m_ of the 8BxHMFO is 0.1 s^−1^ mM^−1^ which compared to the reported *k*_cat_/*K*_m_ value of the double mutant is relatively low (2.2 s^−1^ mM^−1^) (Additional file 8: Figure S3) [11]. This indicates that the higher conversion is largely due to an improved operational stability. The results indicate that this engineered version of HMFO can be used for longer time and can lead in this way to higher amounts of FDCA.

## Conclusions

In this work, we further developed the FRESCO protocol by developing a novel and efficient approach for combining individual stabilizing mutations. The Golden Gate gene shuffling described in this study was fundamental to rapidly identify the best combination of thermostabilizing mutations that led to a stable and active HMFO variant. The engineered 7xHMFO variant presented a *T*_m_^app^ improvement of 12 °C compared with the wild-type enzyme together with an improved cosolvent tolerance. We could also demonstrate that this thermostable variant of HMFO can be used as template to introduce a destabilizing mutation in the active site. One of the resulting HMFO mutants displayed superior performance compared to previously reported HMFO variants in converting HMF in a three-step one-enzyme reaction into FDCA.

## Methods

### Materials

All materials were acquired from Sigma-Aldrich unless otherwise specified.

### Computational methods

The FRESCO method was employed to obtain a thermostabilized variant of HMFO. Computational modeling was performed using the 4UDQ X-ray structure of HMFO (1.6 Å resolution). To avoid mutations that could interfere with the active site, only residues that were > 5 Å away from FAD were mutated [11]. The computational selection was started with calculating the predicted change of free folding energy (ΔΔG^Fold^) with FoldX (foldx.crg.es) and Rosetta-ddg (http://www.rosettacommons.org) [18, 19, 31]. For Rosetta, the so-called row-3 protocol (described by Kellogg et al. in row 3 of their table 1) was invoked using the following options: -ddg::weight_file soft_rep_design -ddg::iterations 50 -ddg::local_opt_only true -ddg::min_cst false -ddg::mean true -ddg::min false -ddg::sc_min_only false -ddg::ramp_repulsive false -ddg::opt_radius 8.0 [32]. For FoldX, the used options were --command=BuildModel --numberOfRuns=5. The single point mutations with a predicted ΔΔG^Fold^ < − 5 kJ mol^−1^ were subsequently submitted to MD simulations under Yasara as previously described [18, 19]. The averaged structures from the MD trajectories were visually inspected comparing the variant simulations with the wild-type HMFO while examining backbone and sidechain flexibility, hydrogen bonds, and hydrophobic exposure. This last in silico step is to further reduce the number of potentially thermostable variants to experimentally screen. All FRESCO specific scripts and code are available at https://www.rug.nl/staff/h.j.wijma.

### Genetic engineering

For all the experiments, the His6x-SUMO-HMFO fusion has been used as it has been demonstrated before that the SUMO protein fused at the N-terminus does not affect the activity nor the thermostability of HMFO [8]. The gene single point variants, the double mutant I73V H74Y, the 8AxHMFO, and the 8BxHMFO were obtained from pET SUMO-HMFO or pBAD SUMO-7xHMFO by whole-plasmid PCR with PfuUltra II Hotstart PCR Master Mix (Agilent). Template DNA was cleaved with DpnI (New England Biolabs) for at least 2 h at 37 °C. *Escherichia coli* NEB 10β (New England Biolabs) chemically competent cells were transformed (heat shock at 41 °C for 45 s) and cells plated on 50 μg mL^−1^ kanamycin or 100 μg mL^−1^ ampicillin LB agar plates. All mutations were confirmed by sequencing.

### Golden Gate gene shuffling

The eightfold mutants were obtained using a gene shuffling approach. For this, we designed two synthetic gene versions of *hmfo*: one with 7 modules each containing one mutated region (the first module containing 2 mutations, and the 8 mutations are: I73V-H74Y, Q187E, G356H, V367L, T414K, A419Y, A435E) and the other synthetic gene with the same modules arrangement but without mutations, corresponding to the wild-type DNA sequence. Each module had been designed to be flanked by BsaI recognition sites: NGAGACC at the beginning and GGTCTCN at the end. Moreover, at the beginning of each fragment, the last 4 base pairs (bp) of the previous module are repeated (or the 5′-4 bp of the overhang region of the acceptor plasmid in the case of the first fragment and at the end of the 7th fragment are replicated 4 bp of the 3′-ligation site of the receiving plasmid). The BsaI cutting sites have been chosen to be unique (at least 3 out of 4 nucleotides in the sticky end have to be different) and not palindromic to avoid unwanted ligations (Additional file 9: Gene Sequences). The synthetic genes have been ordered (GenScript) cloned in two pUK57. A derivative of pBAD SUMO vector designed with BsaI cutting site 5′-TGGTngagacc and ggtctcnCTTG-3′) was used as receiving vector. The restriction–ligation reaction was set up in 20 μL volume with the components: pBAD SUMO 3.75 ng μL^−1^, pUC57wt 2.5 ng μL^−1^, pUC578xmutant 2.5 ng μL^−1^, T4 DNA ligase buffer (Promega), T4 DNA ligase 1.5 U μL^−1^ (Promega), BsaI 1 U μL^−1^ (New England Biolabs); the thermocycler program was: incubation at 37 °C for 5 min and at 16 °C for 10 min repeated 50 times, followed by a final incubation at 50 °C for 10 min (final digestion) and at 80 °C for 10 min (enzyme inactivation). The restriction–ligation reaction (5 μL) were used to transform 100 μL of chemically competent NEB 10β cells plated on 100 μg mL^−1^ ampicillin LB agar plates. The *hmfo* variants were verified first by colony PCR (DreamTaq Green PCR Master Mix, Thermo Fisher) to determine the inserts size and then by sequencing.

### Large-scale expression and purification

For HMFO expression, a culture was started by inoculating 5 mL of preculture (LB supplemented with 50 μg mL^−1^ kanamycin or 100 μg mL^−1^ ampicillin) in 100 mL TB (Terrific Broth with the same antibiotic). In the case of pET constructs, protein expression was induced at OD_600_ 0.5 with 1.0 mM isopropyl β-d-1-thiogalactopyranoside (IPTG), and with 0.02% l-arabinose in the case of the pBAD SUMO-HMFO construct. The latter was obtained by cloning the HMFO DNA sequence using *Nde*I and *Hin*dIII (NEB) in pBAD SUMO. Cells were grown overnight at 24 °C 135 rpm and harvested at 5000*g* for 10 min at 4 °C. The cells pellet was resuspended in 10 mL of 50 mM Tris HCl pH 8.0 with 150 mM NaCl and sonicated 3″ on 6″ off at 70% amplitude. The enzyme was purified from the cell-free extract as described previously [8].

### Small-scale expression

The single point variants were expressed in *E. coli* BL21(DE3) cells with the pET SUMO-HMFO vector. The cultures were prepared using 600 μL of overnight culture (LB supplemented with 50 μg mL^−1^ kanamycin) to inoculate 5 mL TB containing 50 μg mL^−1^ kanamycin in a 24-well plate. The multi-site variants were expressed with the pBAD SUMO-HMFO vector in *E. coli* NEB 10β cells; the cultures were made by inoculating 200 μL of overnight culture (LB supplemented with 100 μg mL^−1^ ampicillin) in 800 μL of TB (100 μg mL^−1^ ampicillin) in 96-well plate. The cultures were incubated at 37 °C with a shaking at 300 rpm and induced with 1.0 mM IPTG [in case of *E. coli* BL21(DE3) cells carrying the pET SUMO-HMFO vector] or with 0.02% l-arabinose (*E. coli* NEB 10β cells carrying the pBAD SUMO-HMFO vector) at an optical density at 600 nm (OD_600_) of 2.0. Expression continued overnight at 24 °C with shaking at 550 rpm.

### Small-scale purification

Cells were harvested by centrifugation at 2250*g* for 20 min at 4 °C. The cell-free extract was obtained after cell lysation: the cell pellet was solubilized in 200 μL lysis buffer (lysozyme 1 mg mL^−1^, deoxyribonuclease I 0.5 mg mL^−1^, MgCl_2_ 10 mM in 50 mM Tris HCl pH 8.0). The solubilized pellet was incubated for 30 min at 25 °C (shaking at 550 rpm), and then, it was frozen in liquid nitrogen and centrifuged at 2250*g* for 45 min at 4 °C. The soluble fraction was filtered (Whatman UNIFILTER 96-well Microplate, GE-Healthcare) at 7*g* for 15 s at 4 °C and mixed with 100 μL of pre-equilibrated Ni-Sepharose resin (GE-Healthcare) for 15 min using an AcroPrep Advance 1 mL 96-well plate (Pall). The flow through was removed and the column was washed 2 times with 200 μL of 50 mM Tris HCl pH 8.0 with 150 mM NaCl, and one time with the same buffer containing 5 mM imidazole. The protein was eluted with 100 μL of 50 mm Tris HCl with 150 mM NaCl containing 500 mM imidazole. The eluate was desalted in 50 mM phosphate buffer pH 8.0 using PD MultiTrap G-25 plates (GE-Healthcare).

### Thermostability assay

The melting temperature of HMFO cell-free extract or purified variants was tested by the ThermoFAD method, which allows to determine the unfolding temperature based on the release of the flavin cofactor [8, 24]. This assay was performed using 20 μL of CFE or 20 μL of purified enzyme in 50 mM phosphate buffer at pH 8.0. The ThermoFAD was also used to determine enzyme concentration after the small-scale purification based on the dT/fluorescence value using a calibration line. The calibration curve prepared with several WT concentrations proved that enzyme concentration does not affect the *T*_m_^app^.

### Activity assays

All the activity assays were performed in 50 mM phosphate buffer pH 8.0 at 25 °C. The HMFO mutants I73V, H74Y, Q187E, G356H, V367L, T414K, A419Y, A435E were tested using enzyme activity towards vanillyl alcohol as reported previously [8]. The gene shuffling library was tested with the coupled H_2_O_2_ detection assay: horseradish peroxidase (HRP) (Sigma), 0.004 U μL^−1^, 4-aminoantipyrine (0.1 mM), 3,5-dichloro-2-hydroxybenzenesulfonic acid (1 mM), and HMF (10 mM), measuring at 515 nm (*ε*_515_ = 26 mM^−1^ cm^−1^) the formation of pink product due to H_2_O_2_ production during the oxidation of HMF by HMFO. This HRP coupled assay was also used to determine *k*_cat_, *K*_m_, and the activity after the incubation at 40 °C for HMFO wild type and for the thermostable.

### Product identification

The conversion performed by HMFO WT, HMFO V367R-W466F, HMFO I73V-H74Y-G356H-V367L-T414K-A419Y-A435E (7xHMFO), HMFO I73V-H74Y-G356H-V367L-T414K-A419Y-A435E-W466F (8AxHMFO), HMFO I73V-H74Y-G356H-V367R-T414K-A419Y-A435E-W466F (8BxHMFO), using 5.0 mM 5-(hydroxymethyl)furfural as a substrate were carried out at 25 or 40 °C, with shaking at 1000 rpm. After the conversion, the enzyme was inactivated at 80 °C for 10 min and eliminated by centrifugation. The products were analyzed by high-performance liquid chromatography as described previously [8].

## Additional files

**Additional file 1: Table S1.** ΔT_m_^app^ of the best 17 single mutants. Results of the ThermoFAD assay performed on cell-free extract and purified enzyme.

**Additional file 2: Table S2.** Activity towards vanillyl alcohol of HMFO mutants I73V, H74Y, Q187E, G356H, V367L, T414K, A419Y, and A435E. All the activity assays were performed in 50 mM phosphate buffer pH 8.0 at 25 °C.

**Additional file 3: Table S3.** Multiple mutant alignment of the 9 best performing multiple-mutants resulted from the gene shuffling (results of 96 plate expression and purification system).

**Additional file 4: Figure S1.** Michaelis–Menten graph of WT and 7xHMFO. Kinetic assay performed with HRP peroxidase in 50 mM phosphate buffer pH 8.0 at 25 °C using HMF as substrate.

**Additional file 5: Figure S2.** Oxidation rates of HMF by HMFO wild-type and thermostable 7xHMFO variant after incubation at 40 °C [1 mM] in phosphate buffer 50 mM pH 8.0. The activity test was performed with HRP peroxidase assay using HMF as substrate.

**Additional file 6: Table S4.** Percentages of products formed during the oxidation of 5 mM HMF by 2 μM of enzyme in phosphate buffer 50 mM pH 8.0 at 25 °C in Eppendorf ThermoMixer C while shaking at 1000 rpm. Average values of two experiments (standard deviations were < 27%, with an average standard deviation of 3.5%). Samples with only phosphate buffer, substrate, and WT enzyme where used as control.

**Additional file 7: Table S5.** Percentages of products formed during the oxidation of 5 mM HMF by 2 μM of enzyme in phosphate buffer 50 mM pH 8.0 at 40 °C in Eppendorf ThermoMixer C while shaking at 1000 rpm. Average values of two experiments (standard deviations were < 7%, with an average standard deviation of 1.0%). Samples with only phosphate buffer, substrate, and WT enzyme where used as control.

**Additional file 8: Figure S3.** Michaelis–Menten graph of 8BxHMFO. Kinetic assay performed with HRP peroxidase in 50 mM phosphate buffer pH 8.0 at 25 °C using FFA as substrate.

**Additional file 9: Gene Sequences.** Synthetic gene sequences for WT and 8xHMFO for the Golden Gate gene shuffling.
