# Supplementary material for: Creating a more robust 5-hydroxymethylfurfural oxidase by combining computational predictions with a novel effective library design
Source: Biotechnol Biofuels. 2018 Mar 1;11:56. doi: 10.1186/s13068-018-1051-x (PMC5831843; doi:10.1186/s13068-018-1051-x)
Supplement: Supplementary file 1 — Additional file 1: Table S1. ΔTmapp of the best 17 single mutants. Results of the ThermoFAD assay performed on cell-free extract and purified enzyme. [file 13068_2018_1051_MOESM1_ESM.pdf]

| **Mutant** | **CFE $\Delta T_m^{app}$ (°C)** | **Purified enzyme $\Delta T_m^{app}$ (°C)** |
|---|---|---|
| I73V | 3.5 | 4.1 |
| H74Y | 2.9 | 2.9 |
| G158S | 1.3 | 0.9 |
| H185Y | 0.8 | 0.6 |
| Q187E | 5.0 | 2.6 |
| S340T | 0.4 | 0.9 |
| G356H | 2.4 | 3.9 |
| S365T | 1.5 | 0.9 |
| V367L | 2.0 | 1.4 |
| G404D | 1.8 | 0.6 |
| G404Y | 1.4 | 1.1 |
| K407I | 1.4 | 0.6 |
| A419M | 1.8 | 1.1 |
| A419Y | 1.8 | 1.6 |
| A435E | 2.1 | 1.9 |
| T414K | 2.0 | 1.4 |
| G311A | 1.6 | 1.1 |
