# Supplementary material for: Creating a more robust 5-hydroxymethylfurfural oxidase by combining computational predictions with a novel effective library design
Source: Biotechnol Biofuels. 2018 Mar 1;11:56. doi: 10.1186/s13068-018-1051-x (PMC5831843; doi:10.1186/s13068-018-1051-x)
Supplement: Supplementary file 2 — Additional file 2: Table S2. Activity towards vanillyl alcohol of HMFO mutants I73V, H74Y, Q187E, G356H, V367L, T414K, A419Y, and A435E. All the activity assays were performed in 50 mM phosphate buffer pH 8.0 at 25 °C. [file 13068_2018_1051_MOESM2_ESM.pdf]

**HMFO**	**k_{obs}(s^{-1})**
WT	20.6
I73V	19.3
H74Y	20.3
Q187E	5.4
G356H	6.4
V367L	23.7
T414K	25.7
A419Y	24.4
A435E	16.6
