# Supplementary material for: Creating a more robust 5-hydroxymethylfurfural oxidase by combining computational predictions with a novel effective library design
Source: Biotechnol Biofuels. 2018 Mar 1;11:56. doi: 10.1186/s13068-018-1051-x (PMC5831843; doi:10.1186/s13068-018-1051-x)
Supplement: Supplementary file 3 — Additional file 3: Table S3. Multiple mutant alignment of the 9 best performing multiple-mutants resulted from the gene shuffling (results of 96 plate expression and purification system). [file 13068_2018_1051_MOESM3_ESM.pdf]

I73V H74Y	Q187E	G356H	V367L	T414K	A419Y	A435E	T_m^{app}	$k_{obs}(s^{-1})$
	wt					wt	59	>10
	wt						60	>10
	wt		wt		wt		59.5	>10
	wt				wt		59.5	>10
	wt			wt	wt	wt	57	>10
	wt		wt		wt	wt	59.5	>10
	wt		wt	wt	wt		59	>10
	wt		wt	wt	wt	wt	59	>10
	wt		wt		wt		58	>10
