# Supplementary material for: Creating a more robust 5-hydroxymethylfurfural oxidase by combining computational predictions with a novel effective library design
Source: Biotechnol Biofuels. 2018 Mar 1;11:56. doi: 10.1186/s13068-018-1051-x (PMC5831843; doi:10.1186/s13068-018-1051-x)
Supplement: Supplementary file 4 — Additional file 4: Figure S1. Michaelis–Menten graph of WT and 7xHMFO. Kinetic assay performed with HRP peroxidase in 50 mM phosphate buffer pH 8.0 at 25 °C using HMF as substrate. [file 13068_2018_1051_MOESM4_ESM.pdf]

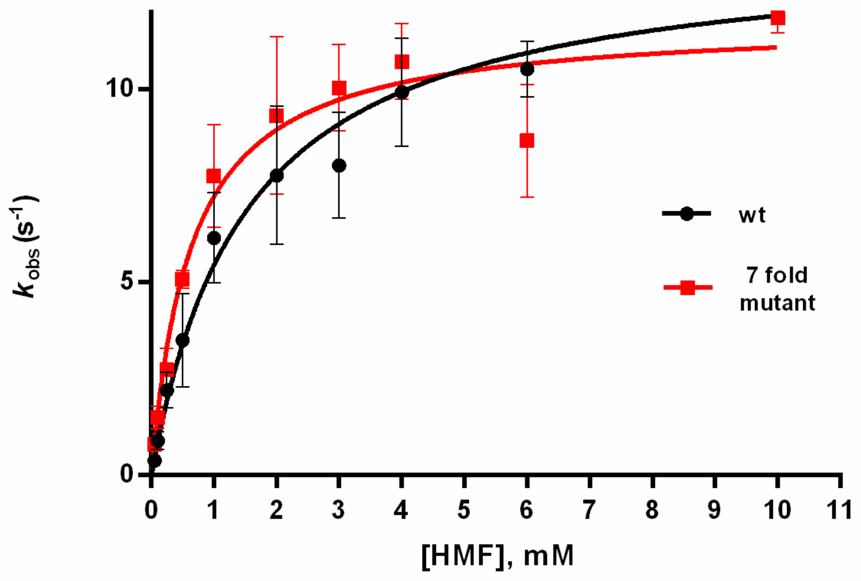

Michaelis-Menten		
**Best-fit values**	**wt**	**7xHMFO**
k_{cat}	13.72	11.79
K_m	1.518	0.6319
**Std. Error**		
k_{cat}	0.70	0.64
K_m	0.24	0.14
**95% Confidence Intervals**		
k_{cat}	12.10 to 15.34	10.30 to 13.28
K_m	0.96 to 2.08	0.30 to 0.96
R^2	0.98	0.96
