# Supplementary figures and images for: Creating a more robust 5-hydroxymethylfurfural oxidase by combining computational predictions with a novel effective library design
Source: Biotechnol Biofuels. 2018 Mar 1;11:56. doi: 10.1186/s13068-018-1051-x (PMC5831843; doi:10.1186/s13068-018-1051-x)

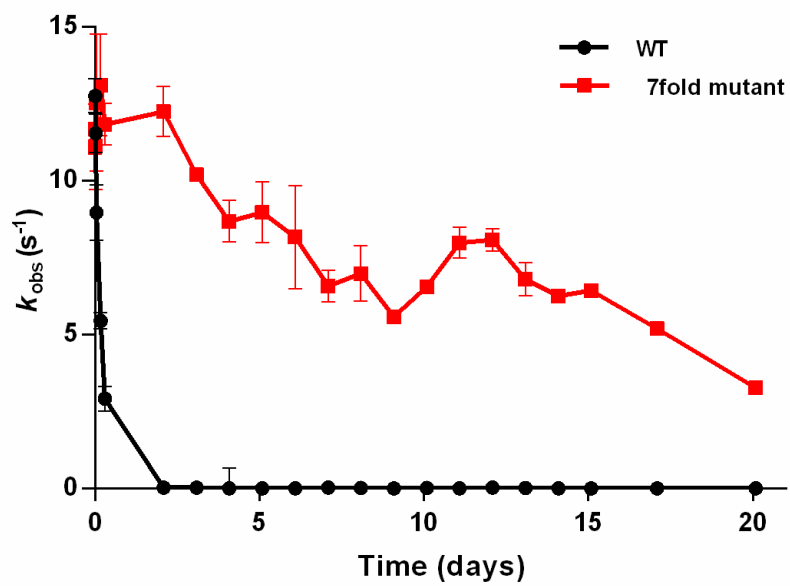

Supplement: Supplementary file 5 — Additional file 5: Figure S2. Oxidation rates of HMF by HMFO wild-type and thermostable 7xHMFO variant after incubation at 40 °C [1 mM] in phosphate buffer 50 mM pH 8.0. The activity test was performed with HRP peroxidase assay using HMF as substrate. [file 13068_2018_1051_MOESM5_ESM.pdf]
