# Supplementary material for: Creating a more robust 5-hydroxymethylfurfural oxidase by combining computational predictions with a novel effective library design
Source: Biotechnol Biofuels. 2018 Mar 1;11:56. doi: 10.1186/s13068-018-1051-x (PMC5831843; doi:10.1186/s13068-018-1051-x)
Supplement: Supplementary file 6 — Additional file 6: Table S4. Percentages of products formed during the oxidation of 5 mM HMF by 2 μM of enzyme in phosphate buffer 50 mM pH 8.0 at 25 °C in Eppendorf ThermoMixer C while shaking at 1000 rpm. Average values of two experiments (standard deviations were < 27%, with an average standard deviation of 3.5%). Samples with only phosphate buffer, substrate, and WT enzyme where used as control. [file 13068_2018_1051_MOESM6_ESM.pdf]

**Reaction time**	**t= 6 hrs**			**t= 12 hrs**			**t= 18 hrs**			**t= 24 hrs**		
**HMFO**	**HMF**	**FFA**	**FDCA**	**HMF**	**FFA**	**FDCA**	**HMF**	**FFA**	**FDCA**	**HMF**	**FFA**	**FDCA**
WT	0.0	79.6	0.7	0.0	92.0	3.0	0.0	100.9	2.3	0.0	98.4	3.1
V367R W466F	54.2	3.4	29.3	20.0	3.9	37.0	40.3	3.2	45.2	35.5	3.1	47.9
8BxHMFO	18.7	2.7	38.4	20.0	3.4	73.0	11.4	1.8	82.6	5.8	0.0	94.1
