# Supplementary material for: Creating a more robust 5-hydroxymethylfurfural oxidase by combining computational predictions with a novel effective library design
Source: Biotechnol Biofuels. 2018 Mar 1;11:56. doi: 10.1186/s13068-018-1051-x (PMC5831843; doi:10.1186/s13068-018-1051-x)
Supplement: Supplementary file 7 — Additional file 7: Table S5. Percentages of products formed during the oxidation of 5 mM HMF by 2 μM of enzyme in phosphate buffer 50 mM pH 8.0 at 40 °C in Eppendorf ThermoMixer C while shaking at 1000 rpm. Average values of two experiments (standard deviations were < 7%, with an average standard deviation of 1.0%). Samples with only phosphate buffer, substrate, and WT enzyme where used as control. [file 13068_2018_1051_MOESM7_ESM.pdf]

Reaction time	t= 3 hrs			t= 6 hrs			t= 9 hrs			t= 12 hrs		
**HMFO**	**HMF**	**FFA**	**FDCA**	**HMF**	**FFA**	**FDCA**	**HMF**	**FFA**	**FDCA**	**HMF**	**FFA**	**FDCA**
WT	69.2	21.8	0.8	64.5	27.9	0.0	62.3	29.2	0.4	60.3	28.8	0.3
V367R W466F	97.6	0.4	0.1	98.7	0.4	0.1	96.8	0.2	0.1	96.1	0.4	0.2
8BxHMFO	22.5	9.2	68.1	9.6	4.6	76.0	7.6	3.9	110.4	1.8	0.0	101.6
