# Supplementary material for: Creating a more robust 5-hydroxymethylfurfural oxidase by combining computational predictions with a novel effective library design
Source: Biotechnol Biofuels. 2018 Mar 1;11:56. doi: 10.1186/s13068-018-1051-x (PMC5831843; doi:10.1186/s13068-018-1051-x)
Supplement: Supplementary file 8 — Additional file 8: Figure S3. Michaelis–Menten graph of 8BxHMFO. Kinetic assay performed with HRP peroxidase in 50 mM phosphate buffer pH 8.0 at 25 °C using FFA as substrate. [file 13068_2018_1051_MOESM8_ESM.pdf]

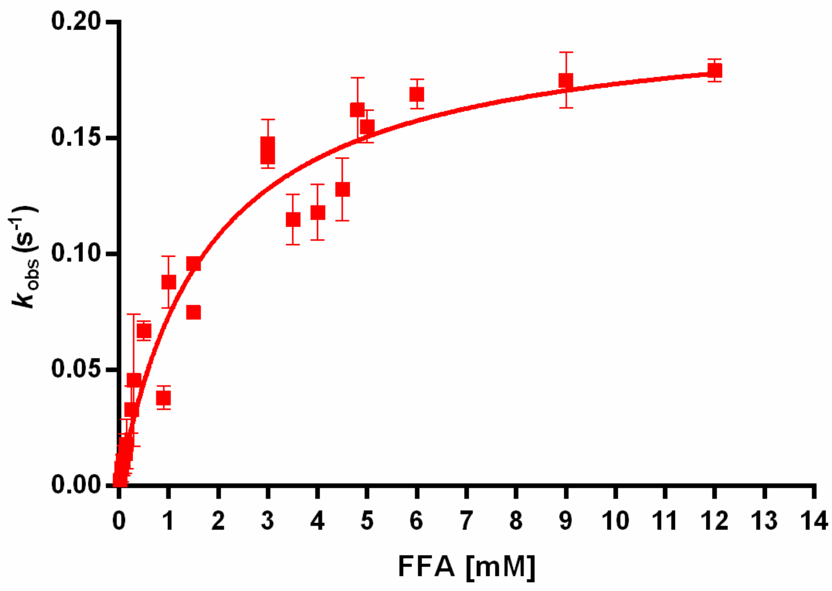

Michaelis-Menten	
**Best-fit values**	**8BxHMFO**
k_{cat}	0.20
K_m	1.78
**Std. Error**	
k_{cat}	0.01
K_m	0.37
**95% Confidence Intervals**	
k_{cat}	0.17 to 0.23
K_m	1.01 to 2.55
R^2	0.94
