# Supplementary material for: Creating a more robust 5-hydroxymethylfurfural oxidase by combining computational predictions with a novel effective library design
Source: Biotechnol Biofuels. 2018 Mar 1;11:56. doi: 10.1186/s13068-018-1051-x (PMC5831843; doi:10.1186/s13068-018-1051-x)
Supplement: Supplementary file 9 — Additional file 9: Gene Sequences. Synthetic gene sequences for WT and 8xHMFO for the Golden Gate gene shuffling. [file 13068_2018_1051_MOESM9_ESM.pdf]

Synthetic genes sequences:

Gene name: HMFO-Wt, Length: 1738 bp,Vector name: pUC57-Kan, Cloning site: EcoRI/HindIII,

BsaI recognition sites
Nucleotides added for the scarless ligation
Sequence:
GAATTCGGTCTCTTGGTATGACTGATACGATTTTTGACTACGTGATTGTTGGCGGTGGCACGGCGGGTAGC
GTTCTGGCCAACCGTCTGTCCGCCCGTCCGGAGAATCGCGTGTTGCTGATTGAGGCCGGTATTGATACCCC
GGAAAACAATATTCCGCCGGAGATCCACGATGGCCTGCGCCCGTGGCTGCCGCGTCTGAGCGGTGATAAGT
TCTTTTGGCCGAATCTGACCATCCACCGTGCCGCGGAACACCCGGGTATCACGCGCGAGCCGCAGTTCTAT
GAACAAGGCCGTCTGCTGGGCGGTGGTAGCAGCGTGAACATGGTCGTTTCTAACCGTGGTCTGCCTCGCGA
CTATGACGAATGGCAGGCACTGGGCGCAGATGGTTGGGATTGGCAGGGAGAGACCGGTCTCTAGGGTGTTC
TGCCGTACTTCATCAAGACCGAGCGTGACGCGGACTACGGTGACGACCCGTTGCATGGCAATGCGGGTCCG
ATTCCGATCGGTCGCGTCGATTCGCGTCACTGGAGCGACTTCACGGTGGCGGCAACCCAAGCTCTGGAAGC
GGCTGGCCTGCCGAACATTCACGACCAAAACGCACGTTTTGATGACGGTTACTTCCCACCGGCATTTACGT
TGAAAGGTGAAGAGCGCTTCAGCGCCGCACGCGGTTATCTGGATGCGAGCGTCCGTGTGCGTCCGAACCTG
AGCCTGTGGACTGAGAGCCGTGTCCTGAAGCTGCTGACCACTGGCAATGCAATCACCGGTGTGAGCGTGCT
GCGTGGTCGCGAAAGAGACCGGTCTCTCGAAACCCTGCAAGTTCAAGCGCGCGAGGTCATCCTGACCGCCG
GTGCGTTGCAAAGCCCAGCGATTCTGTTGCGCACCGGCATCGGCCCTGCGGCGGATCTGCACGCACTGGGT
ATTCCTGTTCTGGCAGACCGTCCGGGTGTTGGTCGCAATCTGTGGGAGCACAGCTCTATCGGTGTGGTTGC
CCCGCTGACCGAGCAGGCACGTGCAGACGCCAGCACGGGTAAAGCCGGCTCTCGCCATCAACTGGGTATCC
GTGCGTCGTCCGGCGTAGATCCGGCGACGCCTAGCGACCTGTTTCTGCATATCGGTGCTGATCCAGTCAGA
GACCGGTCTCTAGTCAGCGGTCTGGCAAGCGCTGTGTTCTGGGTGAACAAGCCAAGCTCCACCGGCTGGCT
GAAGCTGAAGGACGCGGACCCGTTTAGCAGAGACCGGTCTCTTAGCTACCCGGACGTAGACTTCAATCTGC
TGAGCGATCCGCGCGACTTGGGTCGTCTGAAAGCGGGCCTGCGTCTGATCACCCATTACAGAGACCGGTCT
CTTTACTTCGCAGCGCCGTCCCTGGCGAAATATGGTTTGGCGCTGGCATTGAGCCGTTTAGAGACCGGTCT
CTGTTTTGCGGCACCGCAGCCGGGTGGTCCGCTGCTGAACGACCTGTTGCAGGACGAAGCCGCCCTGGAAC
GCTATTTGCGTACGAACGTCGGCGGTGTTTGGCATGCGAGCGGCACGGCGCGTATCGGCCGTGCGGATGAT
TCCCAGGCTGTTGTCGATAAAGCGGGTCGTGTGTACGGCGTCACCGGCCTGCGTGTTGCGGACGCAAGCAT
TATGCCGACCGTTCCGACCGCCAATACCAATCTGCCGACGCTGATGCTGGCTGAGAAAATTGCGGATGCGA
TTCTGACCCAGGCTTAACTTGCGAGACCAAGCTT

Gene name: HMFO-Mut, Length: 1738 bp,Vector name: pUC57-Kan, Cloning site: EcoRI/HindIII,

BsaI recognition sites
Nucleotides added for the scarless ligation
Mutated nucleotides
Sequence:
GAATTCGGTCTCTTGGTATGACTGATACGATTTTTGACTACGTGATTGTTGGCGGTGGCACGGCGGGTAGC
GTTCTGGCCAACCGTCTGTCCGCCCGTCCGGAGAATCGCGTGTTGCTGATTGAGGCCGGTATTGATACCCC
GGAAAACAATATTCCGCCGGAGATCCACGATGGCCTGCGCCCGTGGCTGCCGCGTCTGAGCGGTGATAAGT
TCTTTTGGCCGAATCTGACCGTCTACCGTGCCGCGGAACACCCGGGTATCACGCGCGAGCCGCAGTTCTAT

GAACAAGGCCGTCTGCTGGGCGGTGGTAGCAGCGTGAACATGGTCGTTTCTAACCGTGGTCTGCCTCGCGA
CTATGACGAATGGCAGGCACTGGGCGCAGATGGTTGGGATTGGCAGGGAGAGACCGGTCTCTAGGGTGTTC
TGCCGTACTTCATCAAGACCGAGCGTGACGCGGACTACGGTGACGACCCGTTGCATGGCAATGCGGGTCCG
ATTCCGATCGGTCGCGTCGATTCGCGTCACTGGAGCGACTTCACGGTGGCGGCAACCCAAGCTCTGGAAGC
GGCTGGCCTGCCGAACATTCACGACGAAAACGCACGTTTTGATGACGGTTACTTCCCACCGGCATTTACGT
TGAAAGGTGAAGAGCGCTTCAGCGCCGCACGCGGTTATCTGGATGCGAGCGTCCGTGTGCGTCCGAACCTG
AGCCTGTGGACTGAGAGCCGTGTCCTGAAGCTGCTGACCACTGGCAATGCAATCACCGGTGTGAGCGTGCT
GCGTGGTCGCGAAAGAGACCGGTCTCTCGAAACCCTGCAAGTTCAAGCGCGCGAGGTCATCCTGACCGCCG
GTGCGTTGCAAAGCCCAGCGATTCTGTTGCGCACCGGCATCGGCCCTGCGGCGGATCTGCACGCACTGGGT
ATTCCTGTTCTGGCAGACCGTCCGGGTGTTGGTCGCAATCTGTGGGAGCACAGCTCTATCGGTGTGGTTGC
CCCGCTGACCGAGCAGGCACGTGCAGACGCCAGCACGGGTAAAGCCGGCTCTCGCCATCAACTGGGTATCC
GTGCGTCGTCCGGCGTAGATCCGGCGACGCCTAGCGACCTGTTTCTGCATATCCATGCTGATCCAGTCAGA
GACCGGTCTCTAGTCAGCGGTCTGGCAAGCGCTCTGTTCTGGGTGAACAAGCCAAGCTCCACCGGCTGGCT
GAAGCTGAAGGACGCGGACCCGTTTAGCAGAGACCGGTCTCTTAGCTACCCGGACGTAGACTTCAATCTGC
TGAGCGATCCGCGCGACTTGGGTCGTCTGAAAGCGGGCCTGCGTCTGATCAAACATTACAGAGACCGGTCT
CTTTACTTCGCATATCCGTCCCTGGCGAAATATGGTTTGGCGCTGGCATTGAGCCGTTTAGAGACCGGTCT
CTGTTTTGAGGCACCGCAGCCGGGTGGTCCGCTGCTGAACGACCTGTTGCAGGACGAAGCCGCCCTGGAAC
GCTATTTGCGTACGAACGTCGGCGGTGTTTGGCATGCGAGCGGCACGGCGCGTATCGGCCGTGCGGATGAT
TCCCAGGCTGTTGTCGATAAAGCGGGTCGTGTGTACGGCGTCACCGGCCTGCGTGTTGCGGACGCAAGCAT
TATGCCGACCGTTCCGACCGCCAATACCAATCTGCCGACGCTGATGCTGGCTGAGAAAATTGCGGATGCGA
TTCTGACCCAGGCTTAACTTGCGAGACCAAGCTT
